# Supplementary material for: Quantifying Asymmetric Gait Pattern Changes Using a Hidden Markov Model Similarity Measure (HMM-SM) on Inertial Sensor Signals
Source: Sensors (Basel). 2024 Oct 4;24(19):6431. doi: 10.3390/s24196431 (PMC11479378; doi:10.3390/s24196431)
Supplement: Supplementary file 1 [file sensors-24-06431-s001.zip › sensors-3205581-supplementary.pdf]

Quantifying Gait Pattern Changes using a Hidden Markov Model Similarity Measure (HMM-SM) on Inertial Sensor Signals: Supplementary Materials

Specific p-values from post-hoc t-tests (p-values multiplied by Bonferroni correction for multiple comparisons, adjusted p-values reported in following tables) for each sensor configuration tested in the 3-symmetry-level validity testing. Bolded values indicate statistical significance for that configuration (alpha = 0.05).

Table S1. Pelvis configuration adjusted p-values.

	p-value			
	**2 States**	**3 States**	**4 States**	**5 States**
(1a) $S(SL_1 \| \| SL_2) < S(SL_1 \| \| SL_1)$	**0.007**	**0.015**	**< 0.001**	**<0.001**
(1b) $S(SL_1 \| \| SL_3) < S(SL_1 \| \| SL_2)$	0.077	0.066	0.210	0.067
(1c) $S(SL_1 \| \| SL_3) < S(SL_1 \| \| SL_1)$	**< 0.001**	**0.015**	**< 0.001**	**< 0.001**
(2a) $S(SL_2 \| \| SL_1) < S(SL_2 \| \| SL_2)$	0.225	**0.001**	**< 0.001**	**< 0.001**
(2b) $S(SL_2 \| \| SL_3) < S(SL_2 \| \| SL_2)$	**0.012**	**0.001**	**0.002**	**0.003**
(3a) $S(SL_3 \| \| SL_2) < S(SL_3 \| \| SL_3)$	**0.023**	**< 0.001**	**< 0.001**	**< 0.001**
(3b) $S(S_3 \| \| SL_1) < S(SL_3 \| \| SL_2)$	**0.007**	0.395	0.484	0.400
(3c) $S(SL_3 \| \| SL_1) < S(SL_3 \| \| SL_3)$	**0.003**	**< 0.001**	**< 0.001**	**< 0.001**

Bolded values indicate comparisons which achieved significance threshold (alpha = 0.05)

Table S2. Upper Right configuration adjusted p-values.

	p-value			
	**2 States**	**3 States**	**4 States**	**5 States**
(1a) $S(SL_1 \| \| SL_2) < S(SL_1 \| \| SL_1)$	**0.031**	0.218	**0.009**	**0.006**
(1b) $S(SL_1 \| \| SL_3) < S(SL_1 \| \| SL_2)$	0.233	**0.004**	1.000	1.000
(1c) $S(SL_1 \| \| SL_3) < S(SL_1 \| \| SL_1)$	**0.001**	**< 0.001**	**< 0.001**	**< 0.001**
(2a) $S(SL_2 \| \| SL_1) < S(SL_2 \| \| SL_2)$	1.000	0.178	**0.012**	**0.010**
(2b) $S(SL_2 \| \| SL_3) < S(SL_2 \| \| SL_2)$	0.888	**0.002**	**< 0.001**	**0.004**
(3a) $S(SL_3 \| \| SL_2) < S(SL_3 \| \| SL_3)$	**0.040**	0.088	**0.043**	**0.018**
(3b) $S(S_3 \| \| SL_1) < S(SL_3 \| \| SL_2)$	1.000	0.195	1.000	1.000
(3c) $S(SL_3 \| \| SL_1) < S(SL_3 \| \| SL_3)$	**0.010**	**0.009**	**0.025**	**0.006**

Bolded values indicate comparisons which achieved significance threshold (alpha = 0.05)

Table A3. Upper Left configuration adjusted p-values

	p-value			
	**2 States**	**3 States**	**4 States**	**5 States**
(1a) $S(SL_1 \| \| SL_2) < S(SL_1 \| \| SL_1)$	0.248	0.095	**0.037**	**0.008**
(1b) $S(SL_1 \| \| SL_3) < S(SL_1 \| \| SL_2)$	**0.027**	**0.017**	1.000	0.142
(1c) $S(SL_1 \| \| SL_3) < S(SL_1 \| \| SL_1)$	**0.020**	**< 0.001**	**< 0.001**	**0.003**
(2a) $S(SL_2 \| \| SL_1) < S(SL_2 \| \| SL_2)$	0.140	**0.004**	**0.009**	**0.030**
(2b) $S(SL_2 \| \| SL_3) < S(SL_2 \| \| SL_2)$	**0.038**	**0.006**	**0.012**	**0.001**
(3a) $S(SL_3 \| \| SL_2) < S(SL_3 \| \| SL_3)$	0.277	**0.006**	**0.042**	**0.002**
(3b) $S(S_3 \| \| SL_1) < S(SL_3 \| \| SL_2)$	0.123	0.139	1.000	0.692
(3c) $S(SL_3 \| \| SL_1) < S(SL_3 \| \| SL_3)$	**0.042**	**< 0.001**	**0.017**	**< 0.001**

Bolded values indicate comparisons which achieved significance threshold (alpha = 0.05)

Table S4. Lower Right configuration adjusted p-values

	p-value			
	**2 States**	**3 States**	**4 States**	**5 States**
(1a) $S(SL_1 \|\| SL_2) < S(SL_1 \|\| SL_1)$	**0.017**	**0.032**	**0.032**	0.074
(1b) $S(SL_1 \|\| SL_3) < S(SL_1 \|\| SL_2)$	0.235	1.000	0.119	**0.016**
(1c) $S(SL_1 \|\| SL_3) < S(SL_1 \|\| SL_1)$	**0.002**	**0.005**	**0.011**	**0.003**
(2a) $S(SL_2 \|\| SL_1) < S(SL_2 \|\| SL_2)$	0.068	**0.034**	0.233	0.083
(2b) $S(SL_2 \|\| SL_3) < S(SL_2 \|\| SL_2)$	**0.041**	**0.003**	0.094	**0.006**
(3a) $S(SL_3 \|\| SL_2) < S(SL_3 \|\| SL_3)$	0.075	**0.013**	0.058	0.066
(3b) $S(S_3 \|\| SL_1) < S(SL_3 \|\| SL_2)$	0.814	1.000	1.000	0.216
(3c) $S(SL_3 \|\| SL_1) < S(SL_3 \|\| SL_3)$	**0.026**	**< 0.001**	**0.006**	**0.020**

Bolded values indicate comparisons which achieved significance threshold (alpha = 0.05)

Table S5. Lower Left configuration adjusted p-values

	p-value			
	**2 States**	**3 States**	**4 States**	**5 States**
(1a) $S(SL_1 \|\| SL_2) < S(SL_1 \|\| SL_1)$	0.074	**0.026**	0.123	0.056
(1b) $S(SL_1 \|\| SL_3) < S(SL_1 \|\| SL_2)$	0.064	0.784	0.443	**0.009**
(1c) $S(SL_1 \|\| SL_3) < S(SL_1 \|\| SL_1)$	**< 0.001**	**0.001**	**0.007**	**0.004**
(2a) $S(SL_2 \|\| SL_1) < S(SL_2 \|\| SL_2)$	0.153	**0.018**	0.084	0.055
(2b) $S(SL_2 \|\| SL_3) < S(SL_2 \|\| SL_2)$	**0.005**	**0.045**	**0.011**	**0.034**
(3a) $S(SL_3 \|\| SL_2) < S(SL_3 \|\| SL_3)$	**0.018**	**0.013**	**0.031**	0.290
(3b) $S(S_3 \|\| SL_1) < S(SL_3 \|\| SL_2)$	0.822	1.000	1.000	0.059
(3c) $S(SL_3 \|\| SL_1) < S(SL_3 \|\| SL_3)$	**0.005**	**0.002**	**0.007**	**0.029**

Bolded values indicate comparisons which achieved significance threshold (alpha = 0.05)

Table S6. Upper Right + Upper Left configuration adjusted p-values

	p-value			
	**2 States**	**3 States**	**4 States**	**5 States**
(1a) $S(SL_1 \|\| SL_2) < S(SL_1 \|\| SL_1)$	0.140	**0.011**	**< 0.001**	**0.014**
(1b) $S(SL_1 \|\| SL_3) < S(SL_1 \|\| SL_2)$	**0.005**	**0.012**	1.000	**0.024**
(1c) $S(SL_1 \|\| SL_3) < S(SL_1 \|\| SL_1)$	**0.004**	**0.004**	**0.004**	**< 0.001**
(2a) $S(SL_2 \|\| SL_1) < S(SL_2 \|\| SL_2)$	**0.044**	**0.014**	**< 0.001**	**0.008**
(2b) $S(SL_2 \|\| SL_3) < S(SL_2 \|\| SL_2)$	0.162	**0.010**	**< 0.001**	**0.003**
(3a) $S(SL_3 \|\| SL_2) < S(SL_3 \|\| SL_3)$	**0.010**	**0.001**	**0.009**	**< 0.001**
(3b) $S(S_3 \|\| SL_1) < S(SL_3 \|\| SL_2)$	**0.014**	**0.016**	1.000	0.086
(3c) $S(SL_3 \|\| SL_1) < S(SL_3 \|\| SL_3)$	**< 0.001**	**< 0.001**	**0.002**	**< 0.001**

Bolded values indicate comparisons which achieved significance threshold (alpha = 0.05)

Table S7. Lower Right + Lower Left configuration adjusted p-values

	**p-value**			
	**2 States**	**3 States**	**4 States**	**5 States**
(1a) $S(SL_1 \| \| SL_2) < S(SL_1 \| \| SL_1)$	**0.010**	0.056	0.219	**0.006**
(1b) $S(SL_1 \| \| SL_3) < S(SL_1 \| \| SL_2)$	**0.002**	**0.035**	0.991	**< 0.001**
(1c) $S(SL_1 \| \| SL_3) < S(SL_1 \| \| SL_1)$	**< 0.001**	**0.010**	**0.005**	**< 0.001**
(2a) $S(SL_2 \| \| SL_1) < S(SL_2 \| \| SL_2)$	**0.007**	**< 0.001**	**0.002**	**0.008**
(2b) $S(SL_2 \| \| SL_3) < S(SL_2 \| \| SL_2)$	**0.010**	0.059	0.068	**< 0.001**
(3a) $S(SL_3 \| \| SL_2) < S(SL_3 \| \| SL_3)$	**0.002**	0.071	**0.028**	**0.016**
(3b) $S(S_3 \| \| SL_1) < S(SL_3 \| \| SL_2)$	**0.022**	**0.034**	1.000	**< 0.001**
(3c) $S(SL_3 \| \| SL_1) < S(SL_3 \| \| SL_3)$	**< 0.001**	**0.004**	**< 0.001**	**< 0.001**

Bolded values indicate comparisons which achieved significance threshold (alpha = 0.05)

Figure S1: Example of poor HMM-SM performance

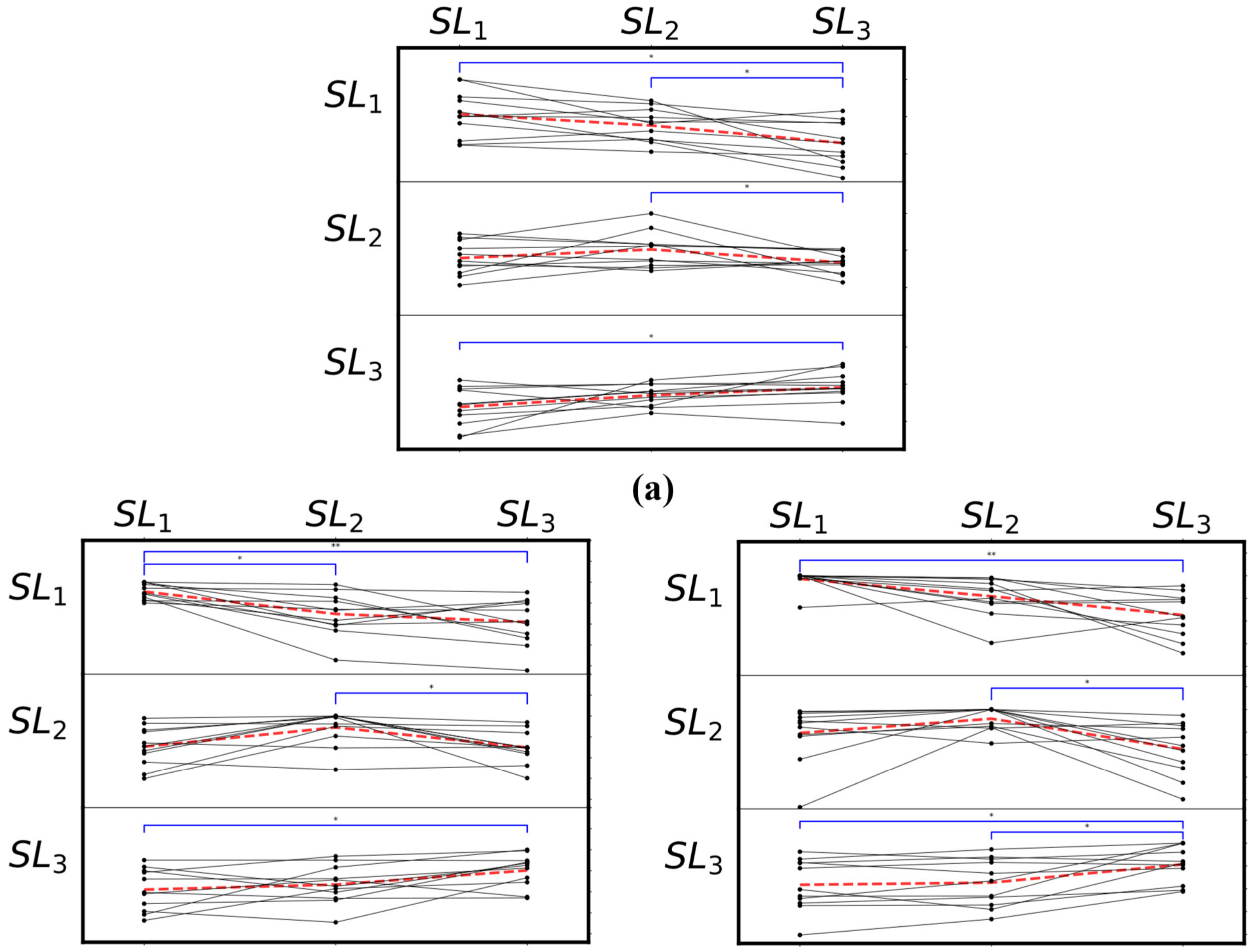

(a)

(b)

(c)

Figure S1: Example of poor HMM-SM performance using results from the 2-state HMMs. Significance bars indicate difference between levels, determined using post-hoc paired t-tests. The red dashed lines indicate the mean response across participants (a) upper left leg, (b) lower right leg, (c) lower left leg.
